# Inkjet-Printing of Nanoparticle Gold and Silver Ink on Cyclic Olefin Copolymer for DNA-Sensing Applications

**DOI:** 10.3390/s20051333

**Published:** 2020-02-29

**Authors:** Martin Trotter, Daniel Juric, Zahra Bagherian, Nadine Borst, Kerstin Gläser, Thomas Meissner, Felix von Stetten, André Zimmermann

**Affiliations:** 1 Hahn-Schickard, Georges-Koehler-Allee 103, 79110 Freiburg, Germany; 2 Hahn-Schickard, Allmandring 9b, 70569 Stuttgart, Germany; 3 Laboratory for MEMS Applications, IMTEK—Department of Microsystems Engineering, University of Freiburg, Georges-Koehler-Allee 103, 79110 Freiburg, Germany; 4 Institute for Micro Integration IFM, University of Stuttgart, Allmandring 9b, 70569 Stuttgart, Germany

**Keywords:** inkjet-printing, gold nanoparticles, electrode integration, DNA sensing, electrochemical sensors, lab-on-a-chip

## Abstract

Inkjet technology as a maskless, direct-writing technology offers the potential for structured deposition of functional materials for the realization of electrodes for, e.g., sensing applications. In this work, electrodes were realized by inkjet-printing of commercial nanoparticle gold ink on planar substrates and, for the first time, onto the 2.5D surfaces of a 0.5 mm-deep microfluidic chamber produced in cyclic olefin copolymer (COC). The challenges of a poor wetting behavior and a low process temperature of the COC used were solved by a pretreatment with oxygen plasma and the combination of thermal (130 °C for 1 h) and photonic (955 mJ/cm²) steps for sintering. By performing the photonic curing, the resistance could be reduced by about 50% to 22.7 µΩ cm. The printed gold structures were mechanically stable (optimal cross-cut value) and porous (roughness factors between 8.6 and 24.4 for 3 and 9 inkjet-printed layers, respectively). Thiolated DNA probes were immobilized throughout the porous structure without the necessity of a surface activation step. Hybridization of labeled DNA probes resulted in specific signals comparable to signals on commercial screen-printed electrodes and could be reproduced after regeneration. The process described may facilitate the integration of electrodes in 2.5D lab-on-a-chip systems.

## 1. Introduction

The introduction of electrodes in lab-on-a-chip cartridges enables various important electrokinetic unit-operations [1] and allows the integration of important electrochemical biosensing methods, which in turn can benefit from the miniaturization, automation, and increased throughput possibilities of microfluidics [2]. The electrodes are commonly produced by screen-printing, etching of printed circuit boards (PCB) or by cleanroom processes like metal evaporation and sputtering [3], often on planar substrates that need to be bonded afterwards to the fluidic layer [2]. Furthermore, these processes are subtractive processes, i.e., the electrode material is extensively deposited and structuring typically requires the production of masks and partially lithography. The electrode material, which is often expensive, is deposited in excess and a majority is waste. Furthermore, the use of masks limits the flexibility for changing layouts.

The digital technology of inkjet-printing is an interesting alternative, as it enables the deposition of functional materials like metal-based nanoparticle inks on a large variety of substrate materials with a high resolution of the printed structures [4,5]. As a mask-less and fully additive printing technology with a short process chain and with a wide variety of available nanoparticle-based inks it is particularly interesting for the cost-efficient fabrication of electronic components [6,7,8] and for various biosensing applications [9,10]. The non-contact process also allows the deposition of functional materials onto 2.5D and 3D surfaces [11,12], e.g., printing into cavities. Gold is a commonly used material for electrodes in biochemical and biomedical applications because of its inertness and the possibility to functionalize it with the well-known thiol-chemistry [13]. By using nanoparticle gold inks, the melting point typically can be lowered to 150 °C and below [14,15]. This allows the fabrication of conductive gold structures on polymer substrates with a relatively low glass-transition temperature, but high resistances [16] or a required post-production activation step [17] demonstrate that challenges still remain.

In this research, we demonstrate for the first time the production of highly conductive, ready-to-use gold electrodes by inkjet-printing of a commercialized nanoparticle gold ink on cyclic olefin copolymer (COC), a typical substrate material for lab-on-a-chip applications thanks to its good chemical resistance, low water absorption and compatibility with biological samples [18].

COC as substrate material with a low glass-transition temperature (≤140 °C for the type used in this work) and its poor wetting behavior are challenging for printing. In this work, the morphology, conductivity and adhesion of a gold electrode, which was inkjet-printed on COC, was characterized in dependency of thermal and photonic sintering parameters, and the optimal process parameters were determined.

The results were used to print electrode arrays for electrochemical nucleic acid hybridization detection. A comparable application scenario has been reported before for inkjet-printed gold electrodes, but on a planar paper substrate [19]. Arrays printed on a planar substrate were first used to characterize the electrochemical properties and electrode functionalization with thiolated capture probes in more detail. The results were compared with commercial screen-printed electrodes. Finally, to demonstrate the possibility of integrating functional electrodes in microfluidic chips, an electrode array was printed into the chamber of an injection molded chip for DNA hybridization detection.

## 2. Materials and Methods

### 2.1. Chemicals, Reagents, and Materials

For the investigations a nanoparticle gold ink from C-INK Co., Ltd. (DryCure Au-J, parts number 1010B, water-based, 10 wt.% Au, Soja City, Japan) and a nanoparticle silver ink from Sun Chemical Corp. (Suntronic EMD5730, ethylene glycol based, 40 wt.% Ag, New Jersey, USA) were used for the inkjet-printing of the conductive structures. Ultraviolet (UV)-curable XP PriElex^®^ SU-8 lacquer from MicroChem Corp. (Westborough, USA) was used to print dielectric passivation layers. The experiments were carried out on injection molded COC substrates. Planar substrates were made from Topas 6013S-04 and substrates containing fluidic chambers from Topas 5013L (Topas Advanced Polymers, Frankfurt, Germany) with a glass-transition temperature of 140 °C and 134 °C, respectively.

The following chemicals were used for characterization and functionalization of the gold structures: Isopropanol (≥99.8%), ethanol (≥99.8%), sodium hydroxide (≥98%), potassium chloride (≥99.5%), sulfuric acid (ROTIPURAN^®^ 95–98%) and double-distilled water (ddDI water), which was used to adjust the concentrations of buffers (unless otherwise stated), were purchased from Carl Roth (Karlsruhe, Germany). Ru(NH_3_)_6_Cl_3_ (98%), 5-mercapto-1-hexanol (97%, MCH), sodium dodecyl sulfate (99%, SDS) and saline-sodium citrate (20 X, SSC) were obtained from Sigma-Aldrich (Steinheim, Germany), Nexterion Spot buffer from Schott (Mainz, Germany), and sodium phosphate buffer from Medicago (Uppsala, Sweden).

Hybridization mixes consisted of Bst 2.0 WarmStart DNA Polymerase, Isothermal Amplification Buffer and MgSO_4_ all purchased from New England Biolabs (Frankfurt, Germany) diluted in DNase/RNase-free water ordered from Life Technologies (Darmstadt, Germany).

All used probes/oligonucleotides were synthesized by Biomers (Ulm, Germany).

### 2.2. Inkjet Printing

Initially, the COC substrates were cleaned in isopropanol combined with an ultra-sonic treatment (Transsonic 420/H from Elma Schmidbauer GmbH, Singen, Germany) for 3 min at 30 °C. Subsequently the substrates were flushed with de-ionized (DI) water and thermally dried in an oven (UF 55^PLUS^ from Memmert GmbH and Co. KG, Schwabach, Germany) at 80 °C for 1 h. A Dimatix DMP 2831 drop-on-demand inkjet-printer from FUJIFILM Dimatix, Inc. with a 10 pl drop size printhead was used. Images of the used waveforms and the printing parameters can be seen in Figures S1–S3 in the supplementary information (SI). The substrate temperature during the printing process of gold and silver ink was set up to 40 °C. Because of the low Au wt.% of the gold ink the Au electrodes were printed multilayered. Nanoparticle inks usually need a sintering step for generating conductive paths. Therefore, a thermal sintering in an oven (UF 55^PLUS^ from Memmert GmbH and Co. KG, Schwabach, Germany) at 130 °C for 1 h was performed. For a further improvement of the conductivity of the printed structures a combination of thermal sintering (1 h/130 °C) and a subsequent photonic curing (energy: 955 mJ/cm², one pulse with 270 V and a pulse duration of 1 s, PulseForge^®^ 1200 (PF) from NovaCentrix, Austin, USA) was performed, too. The suitability of photonic curing of nanoparticle inks on temperature-sensitive substrate materials such as polyester, polyvinyl chloride or polyethylene was reported before [20,21]. COC as a hydrophobic thermoplastic causes a poor wetting behavior of the Au and Ag ink on the substrate surface. For improving the wetting behavior of the inks on the COC substrate, a pretreatment of the COC surface was necessary. A plasma treatment is a common process for improving the wettability of polymer substrate materials. Hwang et al. have reported the improvement of the wetting of COC after a treatment with oxygen plasma [22]. Therefore, a low-pressure plasma (O_2_) was tested. For the low-pressure oxygen plasma treatment a PlasmaPrep_2_ from Diener electronic GmbH + Co. KG (Ebhausen, Germany) was used. The adjustable parameters are the power output, the oxygen flow rate and the processing time. For the optical and electrical characterization, conductive paths with a length of 1 cm were printed with different widths (see SI Figure S4). The influence of the thickness of the conductive paths on the conductivity was investigated by varying the number of printed layers (3 layers, 6 layers and 9 layers). Additionally, squares were printed for the cross cut test (Figure S4B).

Two kinds of inkjet-printed electrode (IPE) arrays were realized. The layout (displayed in SI Figure S5) of 2D IPE arrays was adapted from screen-printed 8X220AT gold electrode arrays (SPE), which were purchased from DropSens (Llanera, Spain). 8 working electrodes (WE) of 2.56 mm diameter, counter (CE), and reference electrodes (RE), each, were printed on a planar COC substrate. Substrate thickness was 1 mm, except for the area where the contact pads were printed. There the thickness was reduced to 0.75 mm to reduce the risk of mechanical abrasion. 2D arrays were used to characterize the properties of inkjet-printed structures for DNA hybridization detection in more detail. A 2.5D array, consisting of 6 WE (1 mm diameter), 1 CE, and 1 RE per chamber, was printed on the bottom of a 0.5 mm deep microfluidic chamber (Figure S6). The traces that connect electrodes and contact pads were printed over the sidewalls of the chambers (45° slope, 0.2 mm edge radii).

The WE and the CE were printed of Au. The RE and contact pads were printed of Ag atop Au structures that serve as adhesion layer. A printed passivation layer of SU-8 ensured that the area of the WE was well-defined for the functionalization step and that traces did not contribute to the measurements e.g., as background signal. Different numbers of Au layers were printed wet-on-wet on the COC substrate for investigating the reachable effective surfaces and the influence on the performance of the 2D IPE array. The electrodes of the 2.5D array featured 6 Au layers.

### 2.3. Characterization of the Printing Process

The measurement of the resistances of the printed conductive paths was carried out by four-wire measurement using a point probe station. The determination of cracks and the measurement of the structure dimensions were done using a microscope. The layer thickness and the cross-sectional area for the determination of the specific resistance were measured using a white light interferometer. The adhesion of the printed structures on the COC substrate was tested by a cross-cut test according to DIN EN ISO 2409:2013. Depending on the amount of the peeled off surface area, a cross cut value can be determined. A cross cut value of 0 means that no surface area at all was peeled off. If more than 65% of the tested surface area is peeled off, the cross cut value will be 5. With this test, the influences of the pretreatment by low-pressure oxygen plasma and the sintering temperature on the adhesion were investigated.

Electrochemical measurements were performed with Interface 1010B potentiostats (Gamry Instruments, Warminster, PA, USA) in noise-reject mode and potentials were recorded vs. an external Ag/AgCl/KCl (sat.) electrode, if not stated otherwise. Cyclic voltammetry (CV) and square wave voltammetry (SWV) were recorded with 2 mV step size at scan-rates and frequencies as indicated in the text. SWV pulse size was set to 50 mV, which corresponds to the peak-to-peak amplitude of the square-wave for this software version (Gamry Framework v7.06). 2D arrays were contacted via a 30 pin card-edge connector delivered with the 8X220AT electrode arrays from DropSens. A custom-made PCB with spring contacts was used to contact the 2.5D array.

The effective surface of the 2D IPE and SPE arrays was determined electrochemically in two ways: first, CVs were performed in deaerated 2 mM Ru(NH_3_)_6_Cl_3_ in 0.1 M KCl between 0.05 V and −0.45 V. The active surface area (A_RuHex_) was derived from the reduction peak of Ru(NH_3_)_6_Cl_3_ using Randles-Sevcik equation [23] assuming a diffusion coefficient of 8.43 × 10^−6^ cm^2^ s^−1^ [24]. Second, CVs were conducted with 200 mV/s in deaerated 0.5 M sulfuric acid between −0.2 V and 1.5 V. The reduction peak (between 1.15 V and 0.7 V) of chemisorbed oxygen was evaluated to determine the surface A_Oxide_ related to the number of gold atoms exposed to the solution assuming a charge density of 390 µC cm^−2^ for an oxygen monolayer [25]. Since the reaction of Ru(NH_3_)_6_Cl_3_ is a fast, diffusion-controlled process, the contribution of possible pores is negligible. A_RuHex_ can thus be a good indicator for the footprint of the electrode, while A_oxide_ includes all the surface in contact with H_2_SO_4_ [26].

### 2.4. Immobilization of DNA Capture Probes

Before functionalization, the electrode arrays were rinsed with isopropanol and DI water. 2D electrode arrays were then cycled in 0.5 M sulfuric acid as described above, afterwards extensively rinsed with DI water and finally dried by flushing with nitrogen. The immobilization solution consisted of 20 µM thiolated capture probes, 1X Nexterion Spot buffer in DNase/RNase-free DI water. We included 20 µM MCH in the immobilization solution according to other reports [27,28]. The immobilization solution was spotted on the WE (3.0 µL and 0.5 µL for electrode diameters of 2.56 mm and 1 mm, respectively) and allowed to react for 2 h in a humid environment. Afterwards, the electrode arrays were rinsed with 1 mM MCH and then incubated in 1 mM MCH for 1 h. Finally, the arrays were washed for 15 min in 1 X SSC and 0.1% SDS at 45 °C, 5 min in 0.1 X SSC and 0.1% SDS at 45 °C, and 5 min in ddDI water at room-temperature. The sensors were dried by flushing with nitrogen and stored in nitrogen atmosphere until use.

Capture probe surface coverage was quantified for 2D electrode arrays comparably to the method established by Steel et al. [29]: Chronocoulometry (stepped from 0.05 V to −0.45 V) was performed in deaerated Tris buffer (adapted to pH 7.4 with HCl) first without and then with 0.15 mM Ru(NH_3_)_6_Cl_3_. To remove the Ru(NH_3_)_6_Cl_3_ after the measurement, the electrode array was immersed for 5 min in 0.1 M sodium phosphate buffer and 1 min in ddDI water.

### 2.5. Electrochemical Detection of Hybridization

The functional immobilization of the thiolated capture probes was approved by hybridization with complementary DNA (signal probe), which carried a methylene blue label at its 5′-end (for sequence see SI Table S1). The hybridization solution consisted of a final concentration of 0.3 µM signal probe solved in amplification reaction buffer, which consisted of 0.8 U/µL Bst 2.0 WarmStart DNA Polymerase, 1 X Isothermal Amplification Buffer, and 6 mM MgSO_4_. The composition was chosen to simulate the conditions of a direct, i.e., without intermediate buffer exchange, end-point amplification analysis (in this case for a Loop-mediated Isothermal Amplification (LAMP) reaction [30]) including all the salts and enzymes that influence or possibly interfere with hybridization.

Hybridization was conducted at room-temperature. The 2D electrode arrays (IPE and the SPE) were assembled in a cartridge to define 8 individual hybridization chambers of 20 µL volume (see SI Figure S7). The array defines the bottom, a silicone gasket the sidewalls, and the lid, which also contained the inlet, was made from poly(methyl methacrylate).

Hybridization was monitored directly after loading of the hybridization solution in the reaction chamber by acquiring SWV and CV in regular periods (each minute during the first 10 min, then each 5 min) for 1 h. Potentials were measured vs. the integrated inkjet-printed or screen-printed Ag RE for IPE and SPE arrays, respectively.

Afterwards, the cartridge was disassembled and the hybridized complementary DNA was stripped by immersing the array in NaOH for 10 min and ddDI water for 1 min. Another hybridization reaction was performed with the recovered electrodes. This time, the complementary signal probe was applied to four electrodes to check reproducibility, while the other four electrodes were challenged with non-complementary signal probes.

The 2.5D array was comparably used. The chamber that contains a volume of 28 µL was defined by sealing with pressure sensitive adhesive 9795R (3M, Neuss, Germany) after electrode functionalization. The electrodes were analyzed every five minutes for one hour. During analysis, the working electrodes were interrogated sequentially as they share one CE and RE.

## 3. Results

### 3.1. Characterization of Inkjet-Printed Structures

#### 3.1.1. Optical Characterization

The wetting behavior on COC was very poor without a pretreatment. The printed structures were full of breaks and it was not possible to generate conductive tracks without a prior surface treatment. This behavior is not unexpected, since the ink is water-based and COC is known to be a hydrophobic material [22].

**Figure 1:**
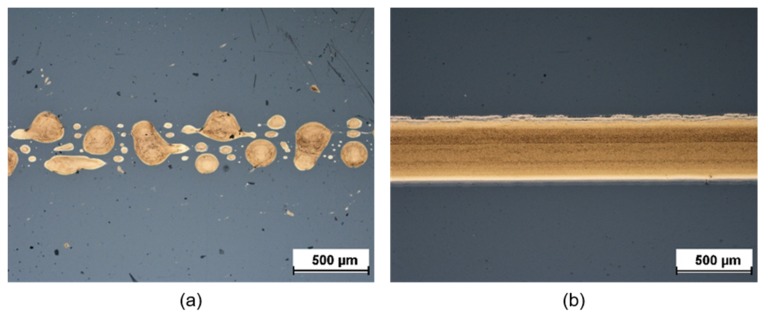
Printing results on cyclic olefin copolymer (COC) without (**a**) and with (**b**) plasma treatment.

The wetting behavior of the Au ink could be significantly improved on COC via a low-pressure oxygen plasma treatment. The best results for COC were obtained with a plasma treatment at 40 W for 20 s and an oxygen flow rate of 280 cm³/min. The corresponding results are shown in Figure 1. It is evident that the acuity of the inkjet-printed structures improved significantly after plasma treatment of the COC substrate. Note that the effect of the O_2_-plasma treatment is only temporary and decreases over time [31], which needs to be taken into consideration for fluidic layout. In this work the COC surface around the printed Au and Ag structures was mostly covered by the SU-8 lacquer.

**Figure 2:**
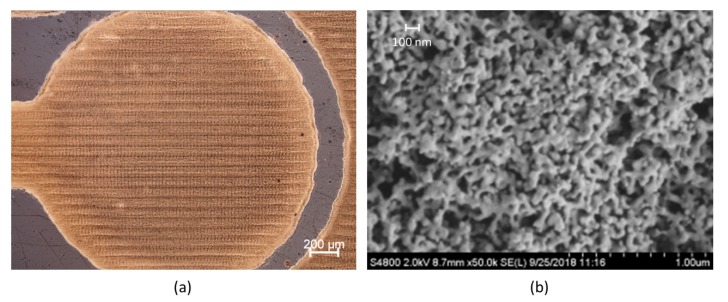
Surface of WE (9 layers) after photonic sintering taken with a reflected light microscope (**a**) and with a SEM (**b**).

Figure 2 shows the micrographs of the printed WE after the photonic curing. The scanning electron microscope (SEM) image of the resulting Au shows a significant formation of sinter necks between the nanoparticles and indicates a porous morphology.

The layer thickness of the printed structures was measured using a white light interferometer. The results showed that the thicknesses were between 200 nm for the 3-layer and 600 nm for the 9-layer printed structures. This results in a thickness of ca. 70 nm per layer.

The widths of the structures in the printing layout were 125 µm (5 pixels (px)), 250 µm (10 px) and 375 µm (15 px). Due to the wetting behavior of the Au ink on the COC, the actual width of the printed structures deviated from the layout. The measured widths of the printed structures were between 260 µm (5 px) and 600 µm (15 px) which results in 60–100% wider structures. The cross-sectional profiles were similar and displayed no visible coffee stain effect.

#### 3.1.2. Electrical Characterization

**Figure 3:**
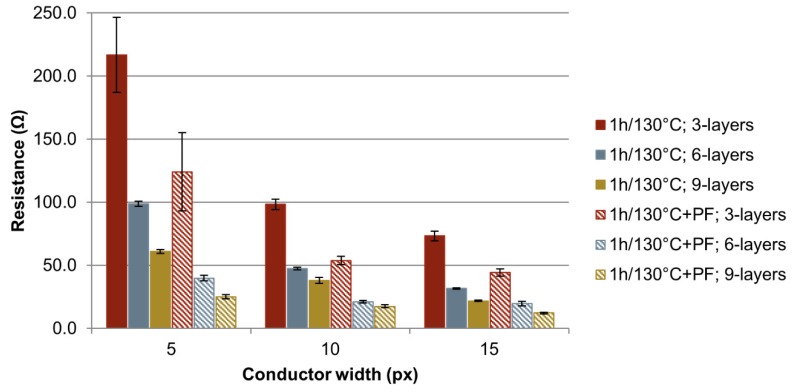
Resistances of printed Au-ink on COC with variation of layer number and sintering parameters. Thermal sintering (1 h/130 °C); Combination of thermal sintering and photonic curing (1 h/130 °C + PF).

Figure 3 shows the variation of the mean resistance of the printed Au-structures sintered for 1 h at 130 °C, which is the highest processable temperature of the used COC. For each mean value a minimum of 18 measurements was carried out. For each of the three different tested number of layers (3-, 6- and 9-layers), one COC substrate was used. The error bars indicate the standard deviation of the measurements. As expected, by increasing the conductor width and the number of printed layers the resistance decreases. By increasing the conductor width, a decrease in the standard deviation was observed. This was caused by small defects in the conductor which have a stronger impact on the resistance of conductors with smaller widths. By performing a photonic curing after the thermal sintering, the resistance could be further reduced by about 50%.

Under these conditions, minimum specific resistances of 49.9 µΩ cm by thermal sintering and 22.7 µΩ cm by the combination of thermal sintering and photonic curing on COC were reached. Compared to bulk gold, this is the 22.5-fold specific resistance for thermally sintered electrodes, and the 10-fold specific resistance for thermally and photonically sintered electrodes.

#### 3.1.3. Adhesion Test

**Figure 4:**
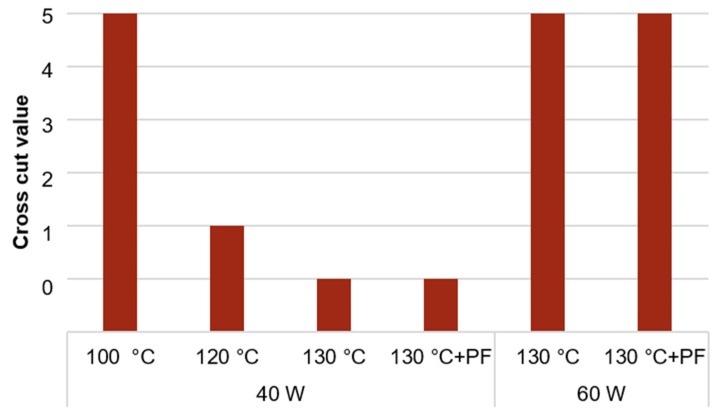
Results of cross-cut test depending on the power of oxygen plasma pretreatment in combination with sintering conditions (temperature without and with subsequent photonic curing (PulseForge^®^ 1200, (PF)).

The results of the cross-cut test and the influence of the sintering temperature and the plasma power on the adhesive strength of the printed structures (6 layers) are shown in Figure 4. For the thermal-sintered Au structures without subsequent photonic curing and a plasma power of 40 W one measurement was carried out. For the remaining values two measurements were carried out, which resulted in the same cross-cut values. With a plasma power of 40 W the adhesive strength of the printed Au structures on COC sintered at 100 °C was poor. By increasing the sintering temperature up to 130 °C, the adhesive strength has increased significantly with an optimum cross cut value of 0. The same result was reached with a thermal sintering at 130 °C and a subsequent photonic curing. However, the structures printed on COC pretreated with 60 W oxygen plasma showed poor adhesive strength. The results showed that the adhesive strength can decrease, if the plasma power is too high. The best results were reached with a plasma power of 40 W for 20 s and a sintering temperature of 130 °C for 1 h with a subsequent photonic curing.

With these parameters further tests were carried out for investigating the influence of water on the adhesive strength and the resistance of the printed gold structure on COC. Electrodes for biosensing are often used in a humid environment where they have to endure these conditions. Therefore, substrates with printed gold structures were immersed in DI water for 1 h. Subsequently the substrates with the printed gold structures were removed from the water and dried with nitrogen. The relative change of the resistances was between −2.3% and 2.4%. There was no indication of a decreasing of the adhesive strength of the printed gold structure measured with the cross-cut test.

In contrast to the Au structures, the adhesion of Ag structures that were directly printed on the COC was reduced when being wetted. Therefore, an Au layer was printed before the Ag as adhesive layer. This is also beneficial because of the much stronger wetting behavior of the Ag ink on the oxygen plasma-treated COC substrate, which caused a much wider spreading of the Ag ink. The Ag ink was sintered thermally at 130 °C for 1 h. Images of the printed electrode arrays are displayed in the SI (Figures S5 and S6)

### 3.2. Inkjet-Printed Electrode Arrays for Electrochemical DNA Detection

#### 3.2.1. Electrochemical Characterization

**Figure 5:**
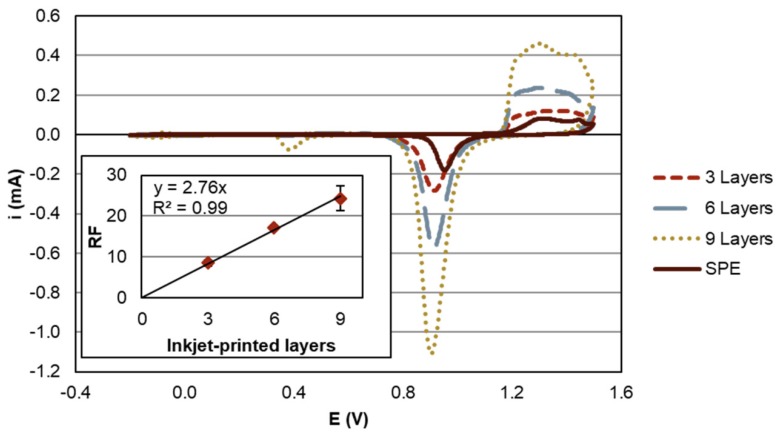
Exemplary cyclic voltammograms of inkjet-printed electrode arrays (IPEs, number of inkjet-printed layers indicated in the legend) and a screen-printed electrode (SPE) in 0.5 M H2SO4. The gold-oxide reduction peak between 1.15 V and 0.70 V was used to calculate the effective surface area. Inset: The calculated roughness factor (RF) in dependence of the number of inkjet-printed layers. The line shows a linear regression fit forced through zero to visualize the correlation of the RF and the printed layers in the investigated range.

The surfaces A_RuHex_ determined by CV in Ru(NH_3_)_6_Cl_3_ differ only slightly for electrodes of different layers and were 4.7 ± 0.1 mm^2^, 4.6 ± 0.1 mm^2^, 6.4 ± 0.6 mm^2^, and 5.0 ± 0.4 mm^2^ for 3-, 6-, 9-layer IPEs and the SPEs, respectively. In contrast, the measurements in sulfuric acid showed a strong dependency on the number of printed layers (Figure 5). A_Oxide_ was 40.8 ± 3.5 mm^2^, 78.5 ± 1.8 mm^2^, and 153.3 ± 9.1 mm^2^ for 3-, 6-, and 9-layer IPEs. When normalizing A_Oxide_ to A_RuHex_ this translates into a roughness factor (RF) of 8.6 ± 0.7, 17.2 ± 0.3, and 24.4 ± 3.0, respectively. The RF is proportional to the number of printed layers (inset of Figure 5). This indicates that the inkjet-printed structures are porous so that the RF can be adapted by the number of inkjet-printed layers.

While the reduction peak established readily for IPEs, indicating a high purity of the gold surface, the SPEs required about 100 cycles in sulfuric acid, until the gold oxide reduction peak fully established (SI Figure S8), which is not unexpected for SPEs and can be attributed to the removal of impurities [32]. A_Oxide_ of SPEs measured after 200 cycles CV was 21.6 ± 3.0 mm^2^, which corresponds to an RF of 4.3 ± 0.3.

#### 3.2.2. Electrochemical Detection of Nucleic Acids

The utility of the IPEs for lab-on-a-chip applications is exemplarily tested for electrochemical nucleic acid hybridization detection. It is of interest whether the low-temperature sintering of the commercial Au ink allows the functionalization of the electrodes, even without a special pretreatment of the surface. To check the functionality and the specificity of the hybridization signal, six of the eight electrodes per array were functionalized with capture probe 1. The other two electrodes served as a negative control and remained non-functionalized, i.e., they were treated like the functionalized electrodes, but the immobilization solution did not contain thiolated capture probes. Furthermore, it is of interest whether the pores of the electrode are accessible for functionalization and hybridization, which may lead to advantageous hybridization signals.

The coverage of electrodes with capture probes scaled with A_Oxide_, which means that the pores are accessible for the capture probes (displayed in SI Figure S9). Surface densities of capture probes referred to A_Oxide_ were 9.8 ± 0.6 pmol cm^−2^, 10.0 ± 0.6 pmol cm^−2^, and 10.2 ± 1.3 pmol cm^−2^, for 3-, 6-, and 9-layered IPEs and 14.1 ± 1.8 pmol cm^−2^ for SPEs. The densities are slightly lower than the previously reported densities of about 15 pmol cm^−2^ for comparable MCH to capture probe ratios, but on planar electrodes and using a different immobilization buffer [27,28].

**Figure 6:**
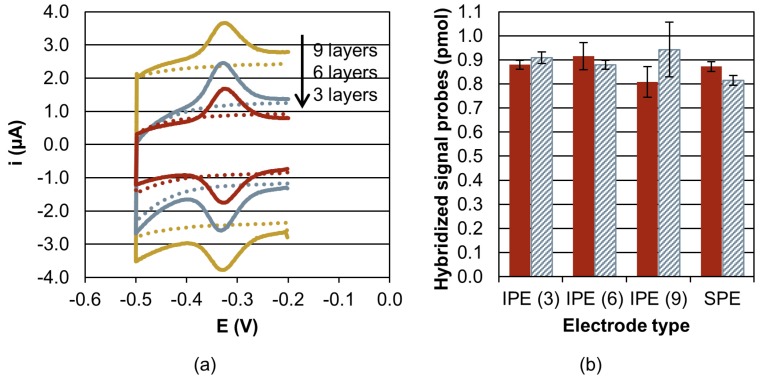
Hybridization signals at electrodes of the 2D arrays. (**a**): Signal recorded by cyclic voltammetry (500 mV/s) vs. the inkjet-printed Ag reference electrode (RE) after one hour of hybridization for functionalized (solid curves) and non-functionalized (dotted) electrodes consisting of 9 (ocher), 6 (grey), or 3 layers (red). For reasons of clarity, the CV of SPE is displayed in the supplementary information (Figure S10). (**b**): Number of hybridized signal probes as calculated from the charge transferred during reduction of the methylene blue label. The result is displayed for the electrodes (3 electrodes per electrode type) that sense complementary signal probes during the first (solid red bars) and the second (hatched grey) hybridization reaction. The number of inkjet-printed layers is given in brackets.

The following hybridization resulted in specific CV signals (acquired in surface mode to account for the surface confined reaction) at the functionalized electrodes (Figure 6a). No CV signals were observed at non-functionalized electrodes, demonstrating that the surface is efficiently blocked against unspecific adsorption of the labelled DNA. The reduction peak was integrated to determine the number of hybridized signal probes (P) based on the charge (Q) transferred from the methylene blue labels according to Faraday’s law P = Q/nF, where F is the Faraday constant and n the number of transferred electrons (n = 2 for methylene blue). The number of hybridized signal probes shows no correlation with the effective surface A_Oxide_ or the number of capture probes (Figure 6b).

After stripping, the rehybridization with the complementary signal probe 1 led to signals that are in good agreement with the first hybridization reaction (Figure 6b), demonstrating a good stability of surface functionalization. Hybridization reactions with the non-complementary signal probe 2 did not result in signals that are detectable by CV.

It is worth noting that the Ag-covered contact pads withstood the seven attachment/detachment cycles with the card-edge connector during the course of the experiment.

Finally, hybridization reactions were conducted with the 2.5D array. Each array featured two electrodes functionalized with capture probe 1, capture probe 2, or no capture probe, respectively. The results (see SI Figure S11) confirmed the specific hybridization of the signal probe exclusively to its complementary capture probes. Furthermore, it proved that electrodes can be successfully introduced in a 2.5D structure.

## 4. Discussion

The inkjet-printed gold-structures presented in this work were stable, when printed on oxygen plasma pretreated COC. The results of the cross cut tests have shown a strong influence of the plasma power and sintering temperature on the adhesion. The strongest adhesion was reached using a plasma power of 40 W for 20 s and a sintering temperature of 130 °C for 1 h with a subsequent photonic curing. A possible reason for the decrease in adhesion of the Au structure on COC could be a damage of the COC surface by the higher ion bombardment with a plasma power of 60 W. A strong overtreatment of the polymer surface can cause an excessive bond breakage and oxidation. This could lead to massive formation of low-molecular-weight oxidized molecules, which remain on the polymer surface as solid debris [33]. When using a lower plasma power of 20 W with a longer processing time up to 60 s of the plasma treatment, the wetting behavior of the Au ink on COC was too poor.

In contrast, the direct printing of Ag on plasma-pretreated COC resulted in structures that delaminated easily when being wetted. The influence of humidity on the adhesion of silver inks on polyimide film (PI) was presented in other works before. The cause of the decreasing of the adhesion was mainly the humidity absorption of the PI [34,35]. In this work COC was used as substrate material, which has much lower moisture absorption than PI. Another possible reason could be an oxidation of the binders and capping agents of the insufficiently sintered silver layer. This could have led to swelling and delamination [34,35]. Printing the Ag ink onto Au structures led to stable Ag structures.

The results show that photonic curing is a convenient alternative for sintering nanoparticle inks. The high energy of the light combined with the short process time enables the sintering of the nanoparticles even on temperature-sensitive substrate materials. The thermal sintering before the photonic curing was performed for removing the solvent from the printed structure. Performing the photonic curing of printed structures with high solvent content can lead to an explosive evaporation of the solvent and cause damage of the printed structures [36].

Photonic curing after thermal sintering at 130 °C yields structures that feature about 10% of the conductivity of bulk gold, which is a 60-fold higher conductivity than previously reported structures on cyclic olefin polymer [16] that were sintered in Ar plasma for 30 min. On other substrates, like polyethylene naphthalate (PEN) or SU-8 covered polytetrafluoroethylene (PTFE) conductivities of a comparable order of magnitude were achieved by thermal sintering at 150 °C [17] and 130 °C [37], respectively. However, the latter sources report the necessity of performing an electrochemical activation step prior to use. This is not required for the electrodes reported by us, as demonstrated for the 2.5D arrays, which were not pretreated prior to functionalization.

Dependent on the sintering parameters, inkjet-printing can result in more or less porous metal structures [38,39]. Apart from few exceptions [40], pores are not desired, since dense structures are typically more stable and conductive. For (bio-)sensing applications, however, an increased surface can be interesting. For example, DNA detection on porous electrodes fabricated by dealloying showed an increased sensitivity [41].

The dependency of A_Oxide_ on the number of inkjet-printed layers clearly shows that the electrodes are porous (Figure 5). The determined number of immobilized capture probes also increases with the number of inkjet-printed layers, which demonstrates that the pores are accessible for capture probe immobilization. As the capture probe density, when referred to A_Oxide_, is comparable for all the IPEs, a homogeneous distribution of the capture probes is likely. However, the amount of hybridized signal probes does not scale with the number of inkjet-printed layers. The lower layers obviously do not contribute to the signal generation within the analysis time (Figure 6b). This may be explained by the reduced accessibility of the pores for signal probes, which is possibly enhanced by electrostatic repulsion of the charged capture and signal probes. This assumption is supported by a study on pore-size restriction for hybridization [42], where pores appear to be accessible for functionalization with capture probes. Access of complementary DNA is strongly dependent on pore diameter and decreases for average pore diameters of about 65 nm. The inkjet-printed structures of this work feature pore sizes in a comparable dimension and below (Figure 2b). Further work would be required to investigate whether an optimization of capture probe density, the use of neutral charge peptide nucleic acid probes [43] or the tuning of pore size e.g., by electrochemical coarsening [42] improves accessibility and hybridization efficiency.

Apart from that, the hybridization signal of IPEs is good and the performance of the IPEs comparable to the commercial SPEs (Figure 6b). In particular, SWV measurements (discussed in SI and displayed in Figure S12) are sensitive to detect and monitor hybridization from the first minute of hybridization. The good reproducibility of electrochemical signals after stripping demonstrates the good stability of the electrodes and their functionalization. Non-specific signals have not been detected, either for electrodes that were functionalized with non-complementary capture probes or for MCH blocked surfaces. This demonstrates the principal suitability of IPEs for DNA-sensing applications and the used conditions with amplification buffer should allow a facile adaption for real applications. As the results indicate that the higher number of porous layers does not contribute to the hybridization signal, the use of 3-layered electrodes is advantageous in terms of a reduced capacitive background and a fast and resource-efficient production process. Compared to the tested SPEs, an activation step (CV in H_2_SO_4_) prior to functionalization was not necessary.

The results for the 2.5D chip (SI Figure S11) successfully demonstrate the integration of electrodes in the fluidic chip over the sidewalls of the chamber, if they feature a slope of 45° and no sharp edges. This enables the facile assembly of electrochemical lab-on-a-chip systems that can be sealed with comparably simple methods like pressure-sensitive adhesives. The commonly used approach [2,44] of bonding a separate fluidic layer to the planar electrode layer becomes then obsolete.

## 5. Conclusions

A commercially available nanoparticle gold ink was deposited with a drop-on-demand inkjet-printer on COC. Because of the low sintering temperature of the gold ink, conductive structures could be realized on COC. For improving the wettability of the gold ink on COC a low pressure oxygen plasma treatment was required. The adhesive strength of the gold ink could be improved by increasing the sintering temperature on COC. The plasma power has shown an influence on the adhesive strength, too. The resistance could be reduced by performing a photonic curing after the thermal sintering. The best results were reached with a plasma power of 40 W for 20 s and a sintering temperature of 130 °C with a subsequently performed photonic curing (Energy: 955 mJ/cm²). With the optimized process parameters, an electrochemical biosensor was successfully realized. The biosensor contained a working and counter electrode printed of gold. The reference electrode was printed of silver. Due to the poor adhesion of the printed silver layer, a gold layer was printed as the adhesion layer.

Analysis of the effective surface revealed that the resulting structures are porous. Even though the porosity of the electrodes had no enhancing effect on DNA sensing in this work, other applications (e.g., glucose sensing [45]) may benefit from the relatively simple production process, which can be an attractive alternative to common fabrication methods like dealloying, template based synthesis [45] or chemical conversion of inkjet-printed Ag structures, which leads to only weakly cohesive porous gold [46].

The final DNA hybridization experiments demonstrate the principal applicability of inkjet-printed electrodes for the specific electrochemical detection of labelled DNA probes in a typical concentration and reaction buffer as used for nucleic acid amplification. Signals are comparable to those generated with commercial SPEs, but IPEs feature the advantage of a reduced pretreatment procedure. The printing of electrodes in a fluidic chamber as demonstrated should facilitate and cheapen the integration of electronics in microfluidic applications.

## Supplementary Materials

The following are available online at https://www.mdpi.com/1424-8220/20/5/1333/s1: Figure S1: Waveform of the Suntronic EMD5730; Figure S2: Waveform of the DryCure Au-J; Figure S3: Waveform of the XP PriElex^®^ SU-8; Figure S4: Printing layouts for the optical and electrical characterization; Figure S5: Design of the 2D electrode array; Figure S6: Schemes and photography of the 2.5D array; Figure S7: Cartridge for hybridization experiments with inkjet-printed and screen-printed 2D arrays; Figure S8: Dependency of A_oxide_ on the number of cyclic voltammetry (CV) cycles in H_2_SO_4_; Figure S9: Number of immobilized capture probes plotted versus the effective electrode area A_oxide_; Figure S10: Exemplary CV signal of functionalized 3-layer IPE and SPE; Figure S11: Hybridized signal probes on 2.5D arrays; Figure S12: SWV signals of the hybridized MB labelled signal probe; Table S1: Oligonucleotide sequences.
