# Supplementary material for: Inkjet-Printing of Nanoparticle Gold and Silver Ink on Cyclic Olefin Copolymer for DNA-Sensing Applications
Source: Sensors (Basel). 2020 Feb 29;20(5):1333. doi: 10.3390/s20051333 (PMC7085783; doi:10.3390/s20051333)
Supplement: Supplementary file 1 [file sensors-20-01333-s001.pdf]

Supplementary Information

Inkjet-Printing of Nanoparticle Gold and Silver Ink on Cyclic Olefin Copolymer for DNA-Sensing Applications

1. Materials and Methods

1.1 Printing parameters

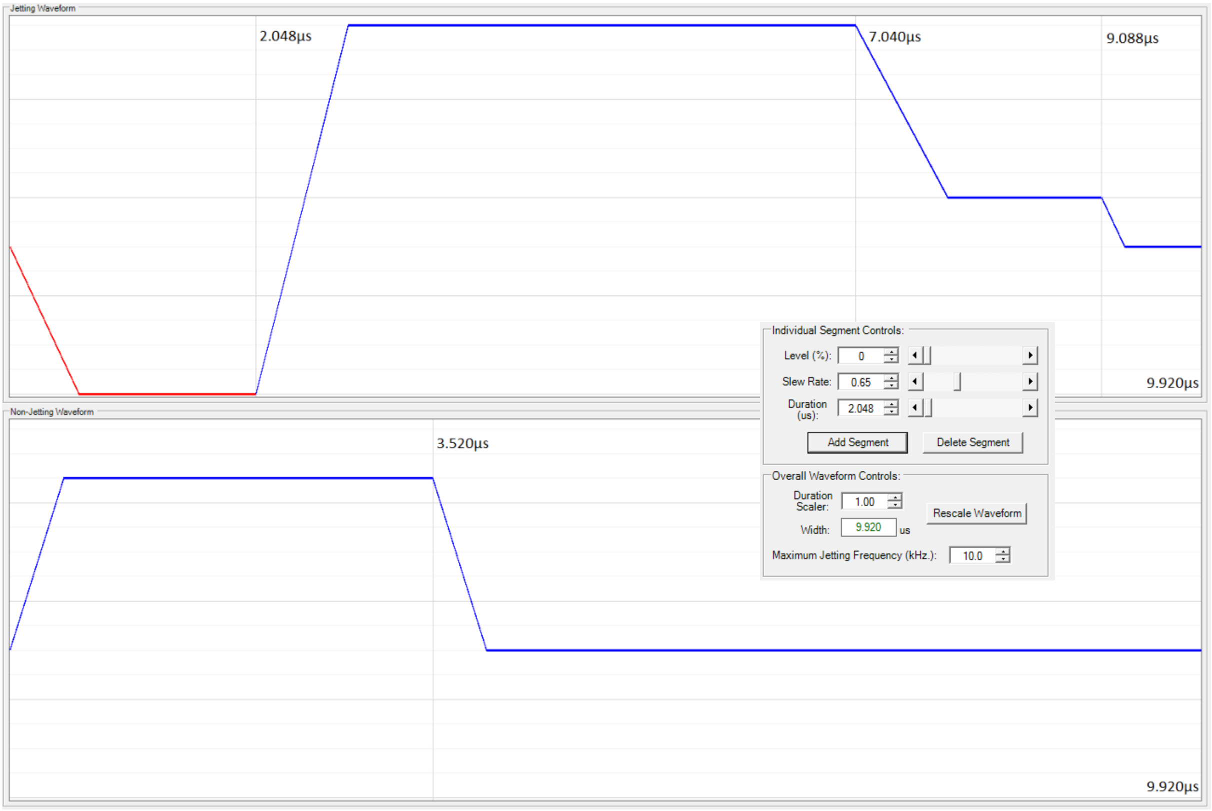

Figure S1: Waveform of the Suntronic EMD5730 (Cartridge temperature: 37 °C, Meniscus vacuum (inches H2O): 4.0, Resolution: 1016 dpi, Substrate temperature: 40 °C)

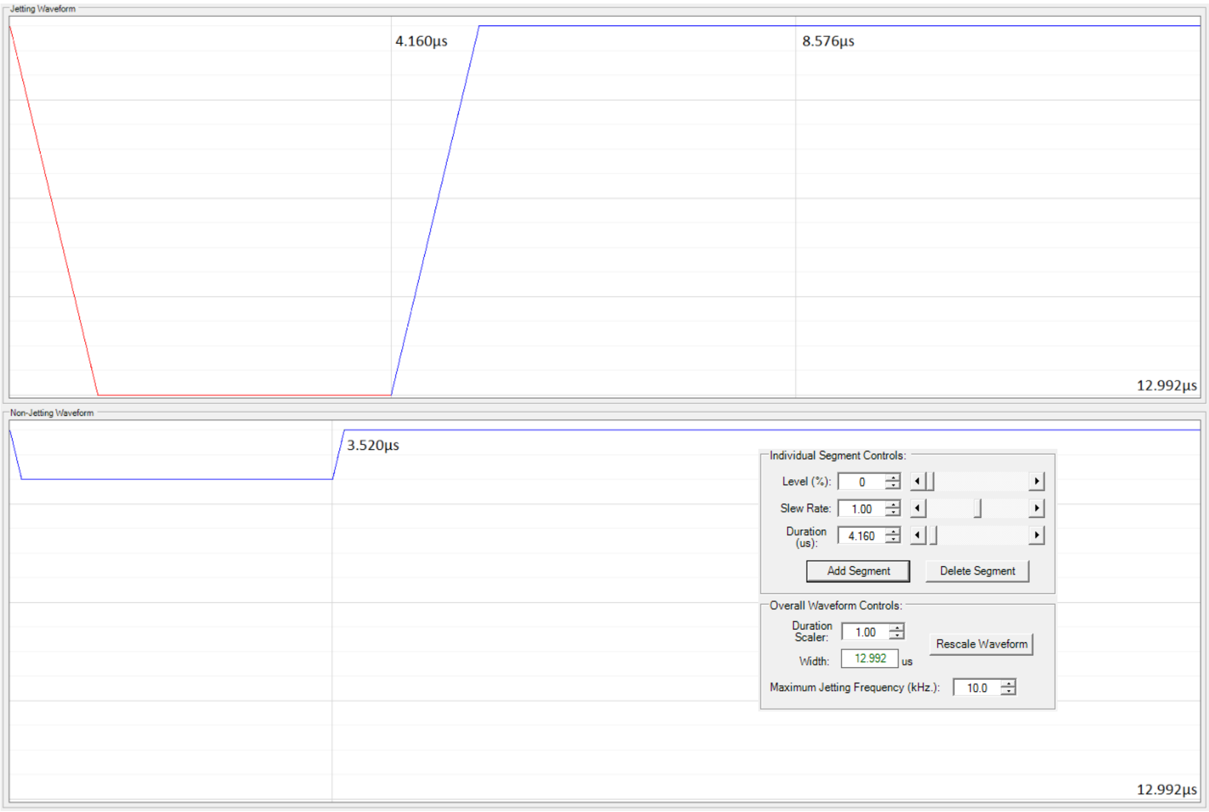

Figure S2: Waveform of the DryCure Au-J (Cartridge temperature: 35 °C, Meniscus vacuum (inches H2O): 5.0, Resolution: 1016 dpi, Substrate temperature: 40 °C)

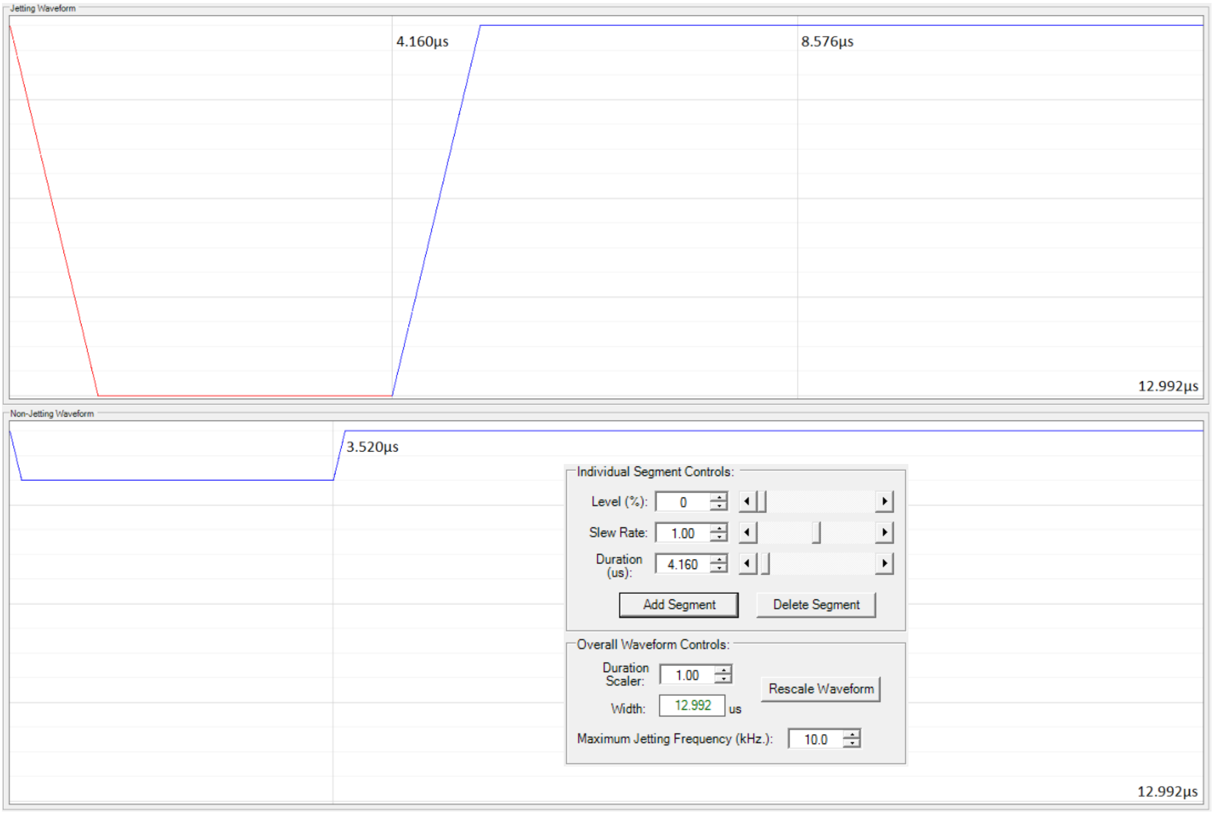

Figure S3: Waveform of the XP PriElex® SU-8 (Cartridge temperature: 45 °C, Meniscus vacuum (inches H2O): 3.0, Resolution: 1016 dpi, Substrate temperature: room temperature)

1.2 Printing layouts

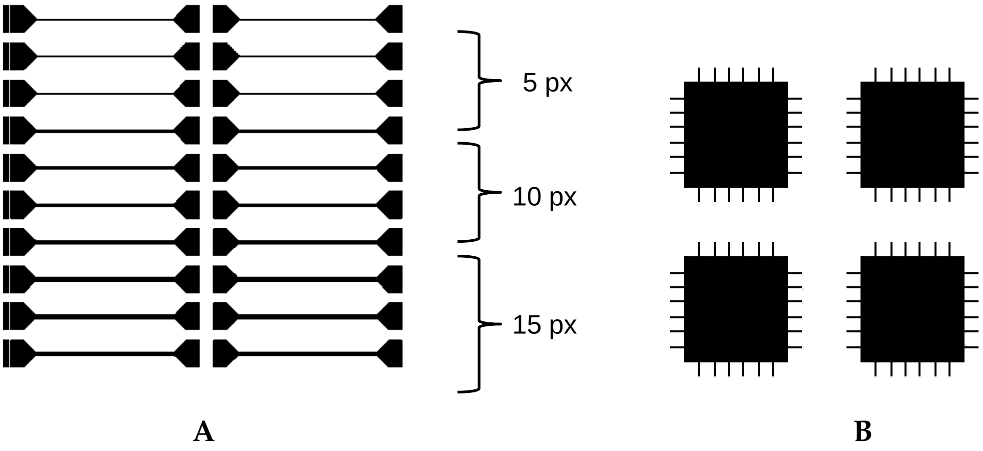

Figure S4: Printing layouts for the optical and electrical characterization (A) and the adhesion test (B). The lines around the squares indicate the position of the cross cuts.

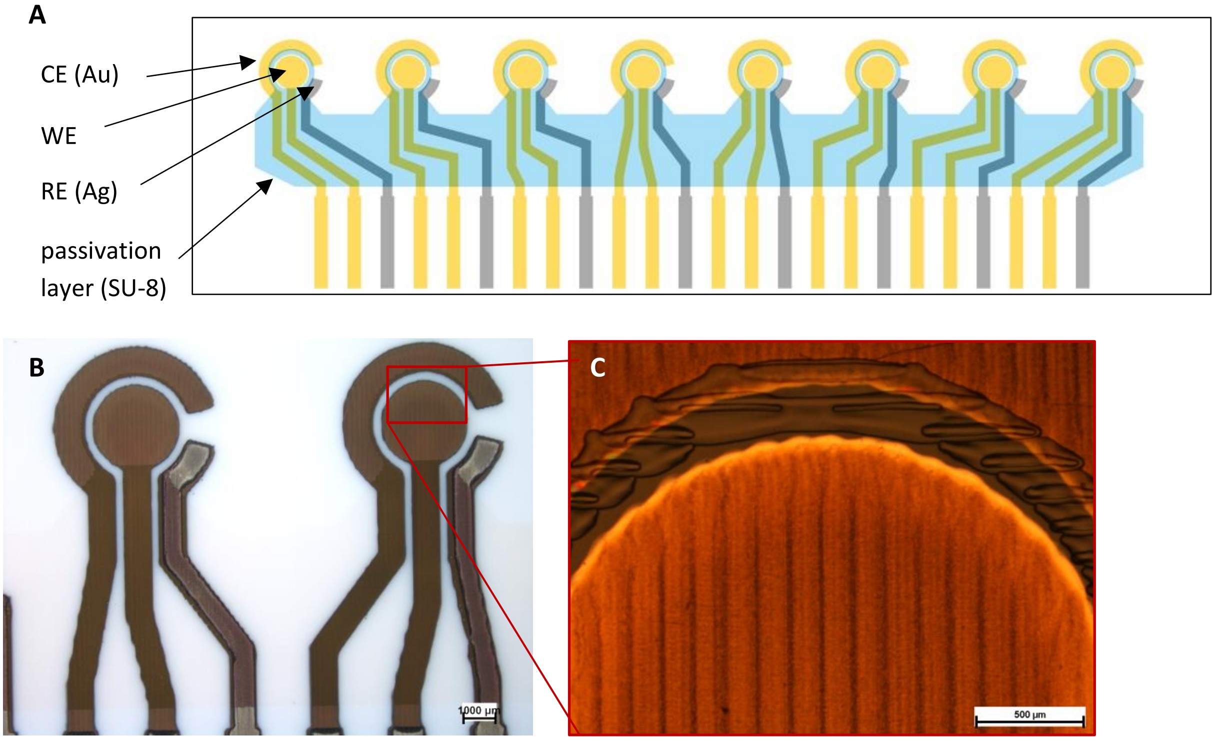

Figure S5: Design of the 2D electrode array (A) and photography of the inkjet-printed result (B). The inspect (C) shows the passivation structure between working and counter electrode. The distance between the electrodes was between 200 and 250 µm.

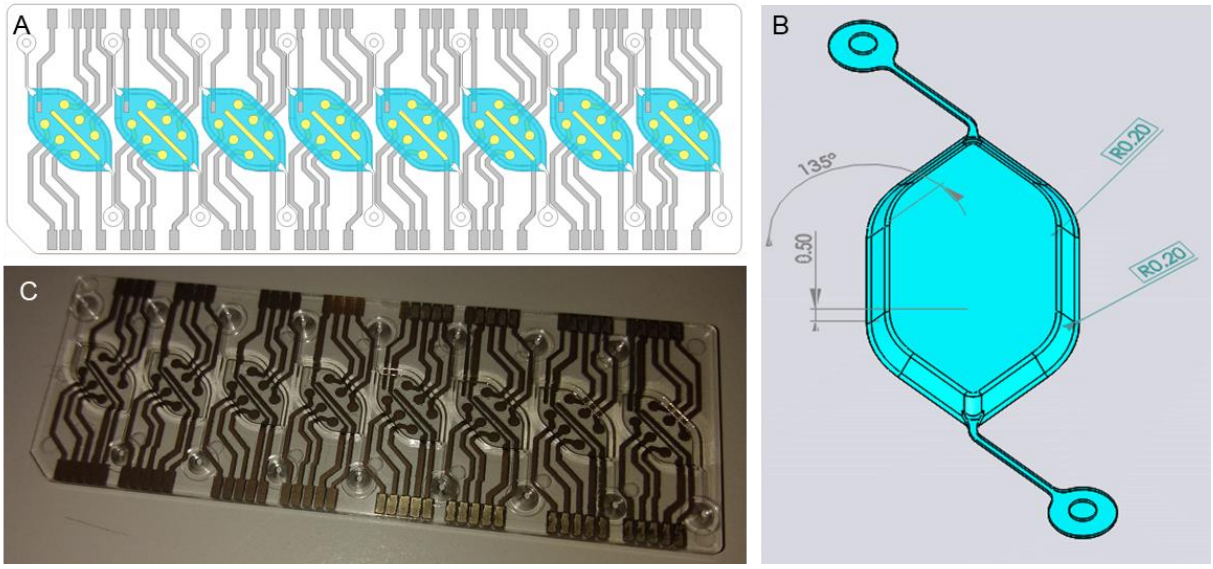

Figure S6: Schemes and photography of the 2.5D array. A: Scheme of the printing layout. Traces and reference electrode were printed with silver atop of gold (grey). Working and counter electrodes were printed with gold only (yellow). Areas covered by the passivation layer are colored in blue. B: 3D sketch of a single chamber featuring the important dimensions for the printing process. C: Photography of the printing result.

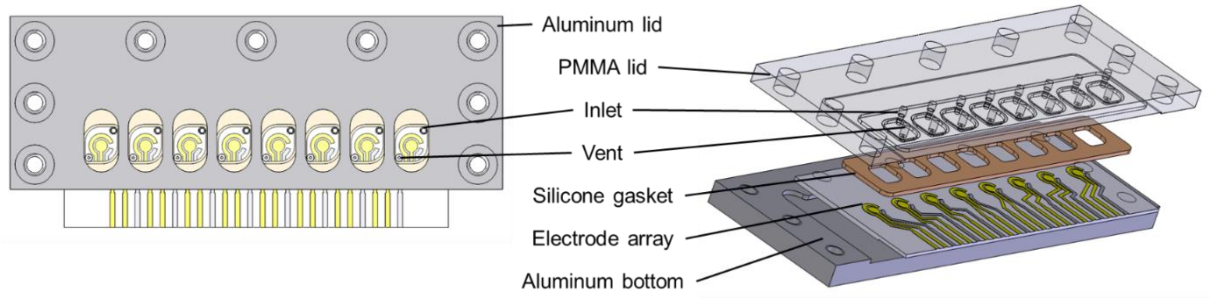

Figure S7: Cartridge for hybridization experiments with inkjet-printed and screen-printed 2D arrays in top-view (left) and exploded view (right). In the area of the contact pads (not covered by the cartridge), the COC substrate was milled on the backside to a final thickness of 0.7 mm in order to reduce the risk of mechanical abrasion caused by attachment/detachment cycles with the connector.

1.3 Oligonucleotide sequences

Table S1: Oligonucleotide sequences used in this work. Signal probes carried a methylene blue (MB) molecule at their 3′-end.

Description	Sequence (5′ – 3′)
Capture probe 1	SH-$(CH_2)_6$-CACTGACCGAACTGAGCTCCTGAGGCATGG
Capture probe 2	SH-$(CH_2)_6$-GGTCGTAGAGCCCATTGCGCGATGAGTGG
Signal probe 1	CCATGCCTCAGGAGCTCAGTTCGGTCAGTG-*MB*
Signal probe 2	CCACTCATCGCGCAATGGGCTCTACGACC-*MB*

2. Results

2.1 Effective surface determined in H_2SO_4

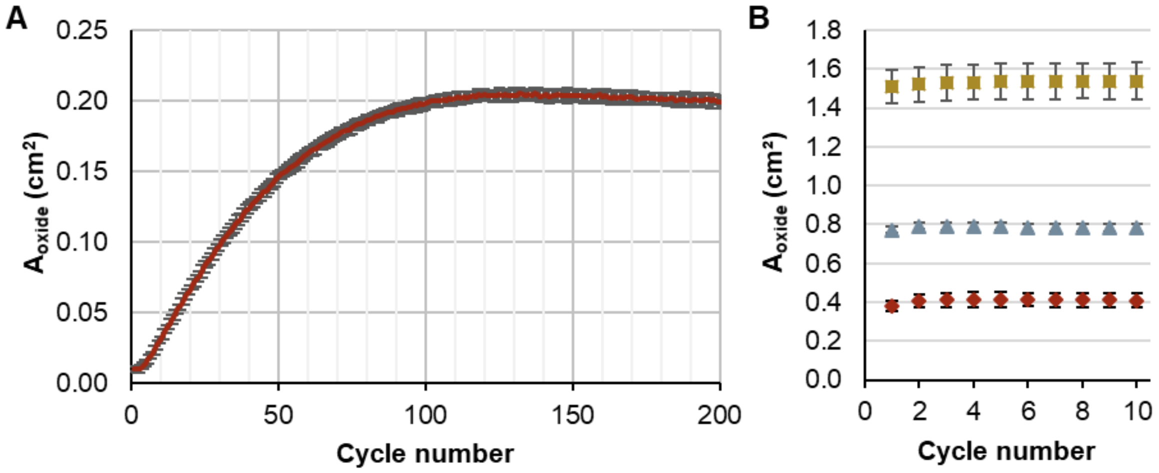

Figure S8: Dependency of A_{oxide} on the number of CV cycles in H_2SO_4 for screen-printed electrodes (A) and inkjet-printed electrodes (B). A: It takes about 100 cycles, until the A_{oxide} (which is proportional to the charge that is transferred during gold oxide reduction) reaches a plateau. B: For 3-layered (red), 6-layered (grey) and 9-layered (ocher) inkjet-printed electrodes A_{oxide} readily establishes, indicating a high purity of the Au surface. The graphs show the means and standard deviations of 8 electrodes for each kind of electrode type.

2.2 Capture probe surface coverage

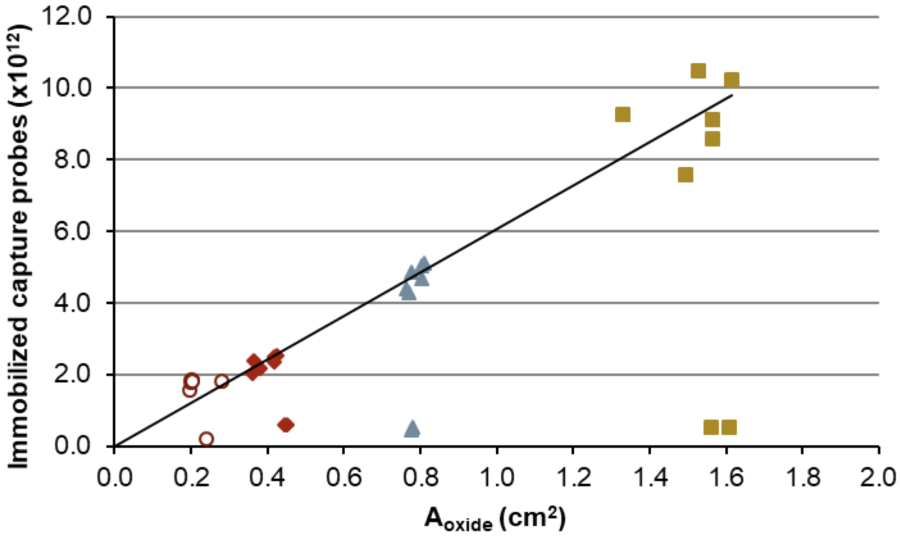

Figure S9: Number of immobilized capture probes plotted versus the effective electrode area A_{oxide}. Values for the screen-printed electrodes, and the 3-, 6-, and 9-layered inkjet-printed electrodes are displayed as open circles, red rhombs, grey triangles, and ocher squares, respectively. The line shows a regression line (forced through zero) for the functionalized electrodes for better visualization of the dependency. Data points below 1 x 10^{12} molecules origin from the non-functionalized electrodes. Since these signals do not scale with A_{oxide}, it is assumed that it is caused by diffusing molecules from the bulk. The data for the functionalized electrodes was not corrected by this value, because the $Ru(NH_3)_6Cl_3$ that is enriched at the capture probes should lower the influence of diffusive or unspecific interactions with the electrode surface due to electrostatic repulsion.

2.3 CV hybridization signal of screen-printed and inkjet-printed electrodes

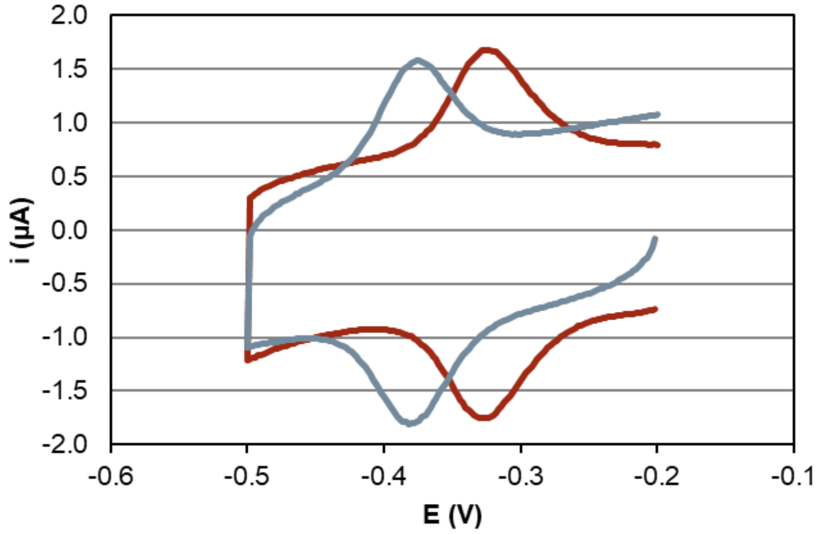

Figure S10: Exemplary CV signal of functionalized 3-layer inkjet-printed electrode (red) and screen-printed (grey) after 60 min of hybridization. The difference in peak potential can be explained by the different composition of the reference electrode.

2.3 Hybridization signal of 2.5D-arrays

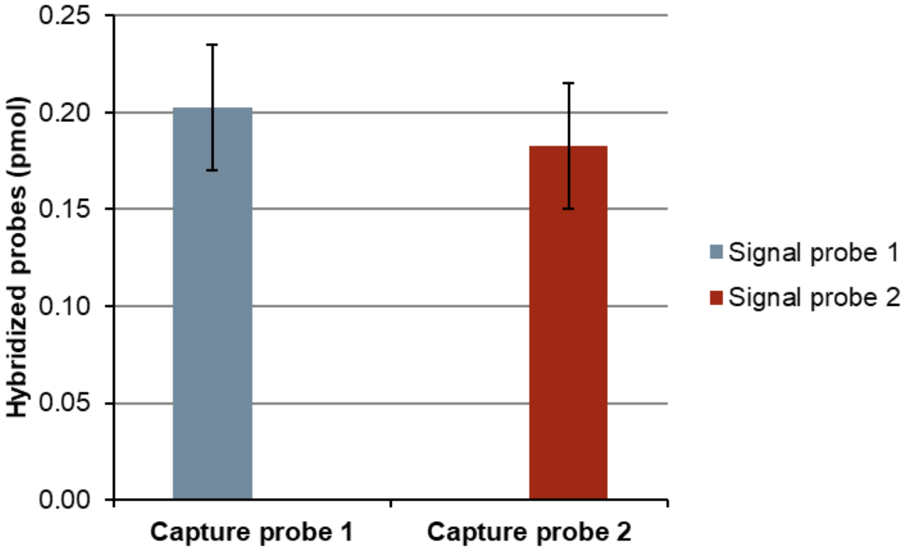

Figure S11: Hybridized signal probes on 2.5D arrays after 60 min of hybridization. Hybridization mixes contained either signal probe 1 or signal probe 2, while each hybridization chamber featured two electrodes functionalized with capture probe 1, two electrodes with capture probe 2 and two electrodes blocked with MCH. Hybridization signals were only detectable at the electrodes functionalized with complementary capture probes. The number of hybridized probes was calculated from the CV reduction peaks of methylene blue as mentioned in the paper. 4 chambers (2 for each kind of hybridization mix) were analyzed. Each bar shows the mean and standard deviation of 4 electrodes.

2.4 SWV signals of 2D-arrays

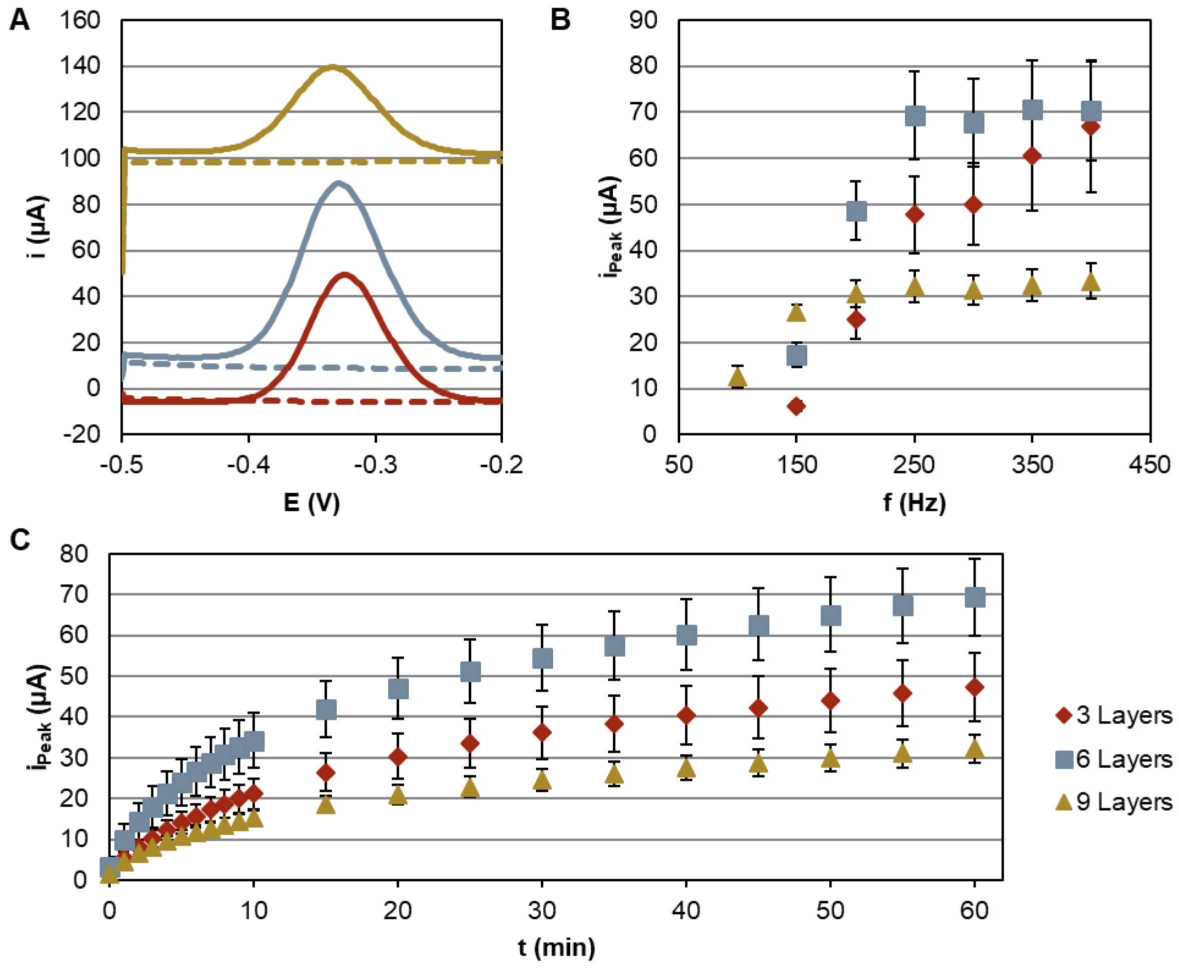

Figure S12: SWV signals of the hybridized MB labelled signal probe. (A) Exemplary SWV curves (250 Hz) after 60 min of hybridization for 3- (red), 6- (grey), and 9-layered (ocher) inkjet-printed electrodes. Among the non-functionalized (dotted lines) and functionalized electrodes (solid lines), only the latter show a hybridization signal. (B) Frequency dependency of the SWV signals for electrodes of different layers. (C) SWV peak currents (250 Hz) for functionalized electrodes in dependence of time. (B) and (C) show the mean and standard deviation of 6 electrodes for each kind of electrode type.

The performance of the different electrode layers depended on the SWV frequency (Figure S12). At 150 Hz, the peak current measured for hybridization at 9-layered electrodes exceeds those of 6- and 3-layered. However, the peak-currents measured in dependency of frequency reach a plateau for 9 layer electrodes at about 200 Hz, and for 6-layer electrodes at about 250 Hz. The 6-layered electrodes outperform those of 3- and 9-layers in the range of 200 Hz to 400 Hz, while the peak currents of 3-layered electrodes steadily increase with frequency and equal those of 6-layered electrodes at 400 Hz - the maximum SWV frequency that can be applied with the Gamry potentiostat. A limiting behavior was also described for other porous structures and attributed to the reduced accessibility of ions [1]. Taking the CV results into account, hybridization reactions towards the lower layers of the pores appear unlikely and thus ion depletion seems to be no satisfying explanation in our case. We assume that the differences in SWV signal instead are related to the different time constants, caused by the capacities of the electric double layer that is naturally higher for larger effective surfaces as

indicated by Figure S12A or also Figure 6A. Besides this issue that requires further analysis, the SWV signals are well detectable and especially helpful to monitor hybridization over time.

3. References

1. Daggumati, P.; Matharu, Z.; Seker, E. Effect of Nanoporous Gold Thin Film Morphology on Electrochemical DNA Sensing. *Anal. Chem.* **2015**, *87*, 8149–8156.
